# Gasdermin E Deletion Attenuates Ureteral Obstruction- and 5/6 Nephrectomy-Induced Renal Fibrosis and Kidney Dysfunction

**DOI:** 10.3389/fcell.2021.754134

**Published:** 2021-10-21

**Authors:** Mengying Wu, Weiwei Xia, Qianqian Jin, Anning Zhou, Qian Wang, Shuzhen Li, Songming Huang, Aihua Zhang, Yue Zhang, Yuanyuan Li, Zhanjun Jia

**Affiliations:** ^1^Nanjing Key Laboratory of Pediatrics, Children’s Hospital of Nanjing Medical University, Nanjing, China; ^2^Department of Nephrology, Children’s Hospital of Nanjing Medical University, Nanjing, China; ^3^Jiangsu Key Laboratory of Pediatrics, Nanjing Medical University, Nanjing, China

**Keywords:** chronic kidney disease, GSDME, pyroptosis, renal fibrosis, inflammation

## Abstract

Renal fibrosis contributes to kidney dysfunction in various chronic kidney diseases (CKDs). Renal fibrosis can be driven by renal tubular cell death and inflammation. Deletion of gasdermin E (*GSDME*), an executor of pyroptosis, has been reported to suppress renal tubular cell pyroptosis in several models of kidney injury. However, additional evidence confirming the role of GSDME in regulating renal fibrosis and kidney function in different CKDs is required. In our study, N-GSDME expression was significantly elevated in CKD models *in vivo* and *in vitro*. *GSDME* deletion alleviated renal fibrosis and inflammation in both unilateral ureteral ligation (UUO) and 5/6 nephrectomy (5/6Nx) models along with the attenuation of renal dysfunction. N-GSDME overexpression had a detrimental effect on fibrotic responses in UUO kidneys and TGF-β1-treated renal tubular epithelial cells. In addition, administration of caspase-3 inhibitor Z-DEVD-FMK, which inhibits caspase-3-mediated GSDME cleavage, protected against renal fibrosis both *in vivo* and *in vitro*. Collectively, these results provide evidence that the activation of GSDME is critical in regulating both renal fibrosis and kidney dysfunction possibly via promoting inflammatory responses in CKD. These findings may offer new insights into the identification of new therapeutic targets for protecting against CKDs.

## Introduction

Chronic kidney disease (CKD) is a common chronic disease with a high prevalence and poor prognosis. CKD has become a global public health problem at present (Levey et al., 2007). Along with increased risk factors such as hypertension, obesity, and diabetes, the prevalence of CKD has increased recently and reached 13.4% worldwide. Patients with apparent kidney function loss always experience end-stage kidney failure owing to limited effective treatments (Hill et al., 2016). Tubule-interstitial fibrosis is widely accepted as the hallmark of CKD. The extent of this pathological process is closely related to the rate of functional decline, which determines the duration of end-stage renal disease (ESRD). Renal fibrosis is centrally implicated in CKD, and is characterized by atrophy of renal tubule epithelial cells and excessive production and accumulation of extracellular matrix (ECM; Schnaper, 2017; Gluck et al., 2019). However, the cellular and molecular mechanisms of renal fibrosis remain unclear. Interventions to prevent or reverse this pathological progression might be important in developing promising clinical treatments for CKD.

Pyroptosis is a type of programmed inflammatory cell necrosis. It is mediated by gasdermin proteins and is dependent on the activity of its specific caspases (Chen et al., 2016). Pore formation on the cell membrane can lead to alterations of osmotic potential, cell swelling, and membrane integrity loss. This results in leakage of cytosolic contents, including some pro-inflammatory factors, which then augments the inflammation cascade reaction (Shi et al., 2015; Ding et al., 2016). Pyroptosis is involved in the progression of various diseases, including immune diseases, neurological diseases, cardiovascular diseases, as well as tumor development (Wu et al., 2016; Mamik and Power, 2017; Lu et al., 2018; Orning et al., 2019). Pyroptosis has also been reported to play crucial roles in contrast-induced acute kidney injury (AKI; Zhang et al., 2018), ischemia-reperfusion kidney injury (Yang et al., 2014), diabetic nephropathy (Li et al., 2017) and lupus nephritis (Peng et al., 2018). Previous studies by our group and others showed that renal tubular epithelial cell pyroptosis and the concomitant enlarged inflammatory responses are critical pathways in the development of AKI (Miao et al., 2019; Li et al., 2020; Xia et al., 2021). Additionally, in the kidney of some CKDs, such as in unilateral ureteral ligation (UUO) and 5/6 nephrectomy (5/6Nx) models, the expressions of NLRP3, caspase-1, and IL-1β, which are key proteins of pyroptosis signaling pathway, are significantly increased (Gong et al., 2016; Guo et al., 2017). However, whether pyroptosis contributes to CKD progression and the specific mechanisms are unclear.

As a member of the gasdermin family that comprises GSDMA, GSDMB, GSDMC, GSDMD, GSDME, and DFNB59, gasdermin E (GSDME) is a newly discovered protein as an executor of pyroptosis. GSDME is highly expressed in normal tissues and can switch tumor necrosis factor-alpha (TNF-α) or chemotherapeutic agent-induced apoptosis to pyroptosis. Similar to GSDMD, an acknowledged implementer of pyroptosis, GSDME has a similar pore forming N-terminus and forms oligomers on the plasma cell membrane, which promotes cell membrane pore formation. Caspase-3 reportedly cleaves the GSDME site specifically at Asp270 (Rogers et al., 2017). Accumulating evidence indicates that GSDME-induced pyroptosis has important implications in various diseases. GSDME-mediated pyroptosis is a pathological mechanism of Adriamycin- induced cardiotoxicity (Zheng et al., 2020). In addition, we previously found that cleavage of GSDME is involved in the process of membrane pore formation and cell lysis of renal tubular epithelial cells and aggravates acute renal tubular injuries (Xia et al., 2021).

The present study was aimed to define the specific role of GSDME in different types of CKD. In this present study, we established two types of CKD mouse model including UUO and 5/6Nx model in GSDME-deficient mice. We also detected the effect of N-GSDME and pharmaceutical inhibitor, Z-DEVD-FMK in UUO model. Our results demonstrated GSDME mediates the initiation of renal fibrosis and kidney dysfunction possibly *via* promoting inflammatory response. Targeting GSDME may be important for the treatment of CKD.

## Materials and Methods

### Animal Studies

Male GSDME^–/–^ mice (8–10 weeks, 25 ± 2 g) were obtained from Professor Feng Shao of National Institute of Biological Sciences. The mice were raised and maintained under a standard specific-pathogen-free conditions with alternating 12 h light and dark cycles and constant temperature and humidity. All mice had unrestricted access to water and food. To study the role of GSDME in UUO kidneys, *GSDME*-knockout (KO) and wild-type (WT) mice were divided into four groups (*n* = 10 per group): WT + sham, WT + UUO, *GSDME*^–/–^ + sham, *GSDME*^–/–^ + UUO. To construct the UUO mouse model, surgery was performed as previously described (You et al., 2020). Briefly, the surgery was performed under aseptic conditions. Mice were anesthetized with 2.0% isoflurane. The left kidney was exposed and 4.0 silk suture was used for ligation of the left ureter. The sham group was conducted without ureteral ligation. Kidney tissues (UUO and sham) were harvested 7 days after surgery for analyses. We also constructed a 5/6Nx mouse model as described previously (Gong et al., 2016). Briefly, *GSDME* KO and WT mice were randomly divided into four groups (*n* = 6 per group): WT + sham, WT + 5/6Nx, *GSDME*^–/–^ + sham, *GSDME*^–/–^ + 5/6Nx. After anesthesia with 2.0% isoflurane, the upper and lower poles of the left kidneys were resected, followed by unilateral right nephrectomy after 1 week. Sham mice were subjected to laparotomy. 8 weeks after 5/6Nx, blood and renal tissues of these mice were collected. Additionally, C57BL/6J mice from the Model Animal Research Center of Nanjing University were divided into four groups (*n* = 8 per group) to examine the role of N-GSDME in tubulointerstitial fibrosis. As described previously (Xia et al., 2021), plasmids (80 μg per mouse) dissolved in saline were quickly injected into the tail vein within 10 s to overexpress N-GSDME *in vivo*. To construct the caspase-3 inhibited animal model, Z-DEVD-FMK [500 ng/mouse/day, the dose used in this study was based on previous report (Xia et al., 2021)] was intraperitoneally injected for 8 days to inhibit caspase-3 expression *in vivo* (pre-treatment of Z-DEVD-FMK for 1 day before UUO operation plus 7-day injection of Z-DEVD-FMK after surgery). All animal protocols were approved by the Nanjing Medical University Institutional Animal Care and Use Committee.

### Masson and Sirius Red Staining

To evaluate the degree of renal fibrosis in each group, kidney tissues of each mouse were obtained and fixed with 4% paraformaldehyde. After dehydration and paraffin embedding, 3 μm thick sections were used for Masson and Sirius Red staining.

### Renal Function and Histology

Blood samples from mice that underwent 5/6Nx was collected and centrifuged at 3000 × *g* for 25 min to obtain serum. Serum creatinine (CR) and blood urea nitrogen (BUN) concentrations were measured by using a serum biochemical autoanalyzer (Hitachi7600 modular chemistry analyzer, Hitachi Ltd., United States) at Nanjing Children’s Hospital.

### Cell Culture and Treatment

The immortalized human tubular epithelial cell line (HK-2) was used to identify the role of GSDME and the underlying molecular mechanisms in renal fibrosis. HK-2 cells purchased from ATCC were cultured in Dulbecco’s modified Eagle medium-F12 supplemented with 10% fetal bovine serum (FBS) (GIBCO, Brazil) in an incubator at 37°C with 5% CO_2_. HK-2 cells were seeded onto six-well plates (6 × 10^5^ cells/well) till they reached 50–70% confluence. The cells were transfected with siRNAs and FL/N-GSDME overexpressing plasmid (2-3 μg/well) or their negative control using Lipofectamine 2000 (Invitrogen, United States). After culturing in a serum-free medium for 5 h, the culture medium was replaced by medium with 10% FBS for 24 h. Different groups of cells were treated with transforming growth factor-beta 1 (TGF-β1, 10 ng/ml) for another 24 h.

### Western Blotting

Cell and kidney tissues were lysed in RIPA lysis buffer (Beyotime, Shanghai, China) containing 2% protease inhibitor (Roche, Switzerland). After incubation for 30 min, proteins in the supernatant were collected after centrifuging at a high speed of 12,000 rpm at 4°C. Protein concentrations were quantified by using a BCA kit (Beyotime, Shanghai, China). Sodium dodecyl sulfate (SDS, Beyotime, Shanghai, China) was added to the cell supernatant before boiling at 100°C for 5 min for protein denaturation. After separation by SDS-polyacrylamide gel electrophoresis and transfer to the polyvinylidene fluoride (PVDF) membrane, the membrane was blocked in 5% non-fat milk for 1 h, followed by incubation with the diluted primary antibodies against fibronectin (FN), alpha-smooth muscle actin (α-SMA), Collagen-1 (COL-1), GSDME, glyceraldehyde 3-phosphate dehydrogenase (GAPDH) and β-actin overnight at 4°C. After washing with Tris–buffered saline with 0.1% Tween^®^ (TBST) three times for 10 min each time, the PVDF membranes were incubated with secondary antibody (A0277, Beyotime, Shanghai, China) for 1 h at room temperature. The signals were visualized using enhanced chemiluminescence.

### Quantitative Real-Time-PCR

Total RNA of cells and kidney tissues was separated using Trizol reagent following the manufacturer’s protocol (TaKaRa Bio Inc., Japan), RNA concentration was determined using OD absorbance at 260/280 nm. RNA (1,000 ng) was reverse-transcribed using a PrimeScript RT Reagent Kit (TaKaRa Bio Inc., Japan). Quantitative PCR was performed using SYBR Green Premix Kit (Vazyme Biotech Co.,Ltd., Nanjing, China) on a QuantStudio 3 real-time PCR system (Applied Biosystems, Foster City, CA, United States). The primers used in this study are listed in Table 1. The mRNA expression levels were normalized to GAPDH and calculated using the 2^–ΔΔCT^ method.

**TABLE 1:** Primer sequences for quantitative real-time-PCR (qRT-PCR).

Gene	Sequence
m-FN	F: ATGTGGACCCCTCCTGATAGT
	R: GCCCAGTGATTTCAGCAAAGG
m-α-SMA	F: GTCCCAGACATCAGGGAGTAA
	R: TCGGATACTTCAGCGTCAGGA
m-COL-1	F: GCTCCTCTTAGGGGCCACT
	R: CCACGTCTCACCATTGGGG
m-TNF-α	F: TCCCCAAAGGGATGAGAAG R: CACTTGGTGGTTTGCTACGA
m-IL-6	F: TAGTCCTTCCTACCCCAATTTCC
	R: TTGGTCCTTAGCCACTCCTTC
m-MCP-1	F: GCTCTCTCTTCCTCCACCAC
	R: ACAGCTTCTTTGGGACACCT
m-GAPDH	F: AAGAAGGTGGTGAAGCAGG
	R: GAAGGTGGAAGAGTGGGAGT
H-FN	F: CGGTGGCTGTCAGTCAAAG
	R: AAACCTCGGCTTCCTCCATAA
H-COL-1	F: GAGGGCCAAGACGAAGACATC
	R: CAGATCACGTCATCGCACAAC
H-GAPDH	F: GGAGCGAGATCCCTCCAAAAT
	R: GGCTGTTGTCATACTTCTCATGG

### Enzyme-Linked Immunosorbent Assay

After homogenization in a plastic collecting pipe, the kidney tissue lysates were collected. Concentrations of IL-1β in the tissue lysates were measured using a mouse IL-1β enzyme-linked immunosorbent assay (ELISA) kit (R&D Systems, Minneapolis, MN, United States) following the manufacturer’s instructions.

### Statistical Analysis

All data are presented as mean ± SEM. Statistical analysis was performed using Student’s *t*-test or analysis of variance on GraphPad Prism (GraphPad, United States). Statistical significance was set at *p* ≤ 0.05.

## Results

### Gasdermin E Cleavage Is Induced in Unilateral Ureteral Ligation Mice Kidneys and TGF-β1 Treated Renal Tubular Epithelial Cells

**FIGURE 1:**
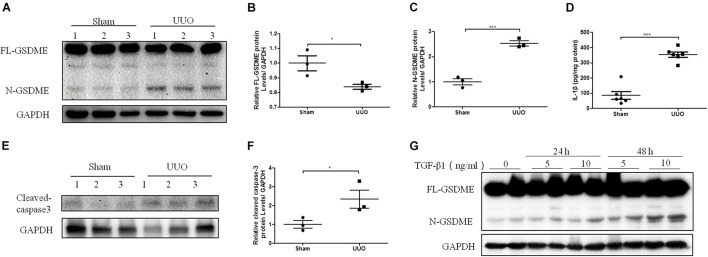
Gasdermin E (GSDME) cleavage is induced in unilateral ureteral ligation (UUO) mice kidneys and TGF-β1-treated renal tubular epithelial cells. Mouse kidney tissues subjected to UUO and human renal epithelial cells treated with TGF-β1 were used to detect the expression of GSDME and pyroptosis. **(A–C)** Western blotting analysis of full length gasdermin E (FL-GSDME) and N-GSDME in kidneys of UUO mice (*n* = 3). **(D)** Secretion of IL-1β in kidneys of UUO mice (*n* = 6). **(E,F)** Western blotting analysis of cleaved caspase-3 in kidneys of UUO mice (*n* = 3). **(G)** Western blotting analysis of FL-GSDME and N-GSDME in HK-2 cells challenged with TGF-β1 at a dosage of 5 or 10 ng/mL for 24 or 48 h. For the cell experiments, two independent experiments were performed. The quantitative results were shown as the means ± SEM. *p < 0.05; ***p < 0.001.

Pyroptosis mediated by gasdermin family members is dependent on the release of its N-terminal domain, which can target the phospholipid membrane and then form the pores to release cellular contents (Ding et al., 2016). Consequently, N-gasdermin formation, the specific caspase activation, and leakage of cytoplasmic contents, including IL-1β, IL-18 and LDH are considered evidence of pyroptosis. To investigate whether GSDME is involved in the progression of CKD, we first determined the expression of full length GSDME (FL-GSDME) and its cleaved form, N-GSDME, in kidneys after UUO for 7 days. N-GSDME protein levels in the UUO group were significantly elevated compared with that in the sham mice, accompanied by the diminution of FL-GSDME (Figures 1A–C). In addition, IL-1β production was greater in UUO kidneys than in the sham group (Figure 1D). Additionally, caspase-3, the enzyme that cleaves GSDME, was excessively activated (Figures 1E,F). These results suggest that GSDME cleaved by caspase-3 might be involved in the development of renal damage after UUO. Furthermore, HK-2 cells were treated with TGF-β1 and N-GSDME expression was determined in this *in vitro* model of renal fibrosis. TGF-β1 induced the production of N-GSDME in HK-2 cells in time- and dose-dependent manners (Figure 1G), which was consistent with the *in vivo* results.

### Gasdermin E Deletion Ameliorates Renal Fibrosis and Inflammatory Responses Induced by Unilateral Ureteral Ligation

**FIGURE 2:**
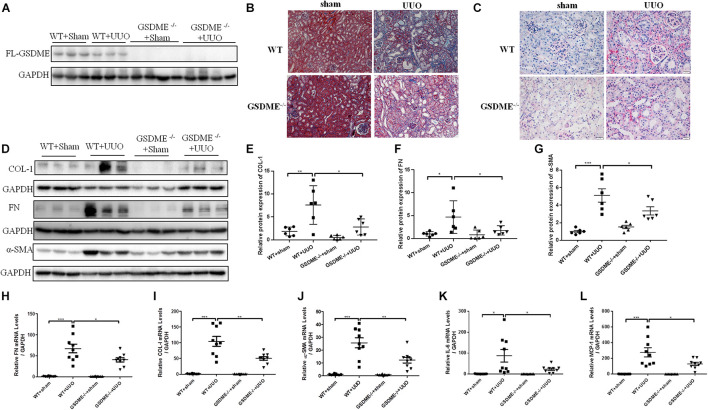
*Gasdermin E* deletion ameliorates UUO-induced renal fibrosis and inflammation. *GSDME* knockout (KO) mice and wild-type (WT) mice were used to define the role of GSDME in UUO-injured kidneys. **(A)** Western blotting analysis of GSDME protein expression in *GSDME* KO kidneys and the controls (*n* = 3–4). **(B)** Masson staining of kidney sections of GSDME KO mice and WT mice (200×). **(C)** Sirius Red staining of *GSDME* KO mice and WT mice (400×). **(D–G)** Western blotting analysis of fibronectin (FN), Collagen-1 (COL-1) and alpha-smooth muscle actin (α-SMA) in mouse kidneys from different groups (*n* = 6). **(H–L)** qPCR analysis of FN, COL-1, α-SMA, IL-6 and MCP-1 in mouse kidneys from different groups (*n* = 9). The quantitative results were shown as the means ± SEM. *p < 0.05; **p < 0.01; ***p < 0.001.

Gasdermin E deletion has been reported to protect mice from chemotherapy-induced acute renal damage (Xia et al., 2021). To determine whether there is also a regulatory effect of GSDME in CKD, *GSDME*-deficient mice were subjected to UUO (Figure 2A). Pathological Masson staining revealed that tubulointerstitial fibrosis was significantly induced in the kidneys of WT mice 7 d after UUO and was significantly ameliorated by *GSDME* deletion (Figure 2B). Collagen fiber staining by Sirius Red also showed that *GSDME*-KO mice displayed less renal collagen deposition than that in WT mice (Figure 2C). GSDME-KO obstructed kidneys also displayed decreased ECM synthesis, as shown by decreased protein and mRNA levels of FN, α-SMA and COL-1 compared with those in the obstructed kidneys (Figures 2D–J). The findings suggested that *GSDME* deletion could protect obstructed kidneys against renal fibrosis. The inflammatory response is involved in the pathogenesis of renal fibrosis in CKD and can further aggravate renal fibrosis. Measurements of the mRNA levels of IL-6 and MCP-1 revealed that the expression of these inflammatory cytokines was remarkably increased in WT-UUO kidney tissues and significantly inhibited in *GSDME* -KO mice (Figures 2K,L).

### Full Length-Gasdermin E Promotes Profibrotic Response in TGF-β1-Treated HK-2 Cells

**FIGURE 3:**
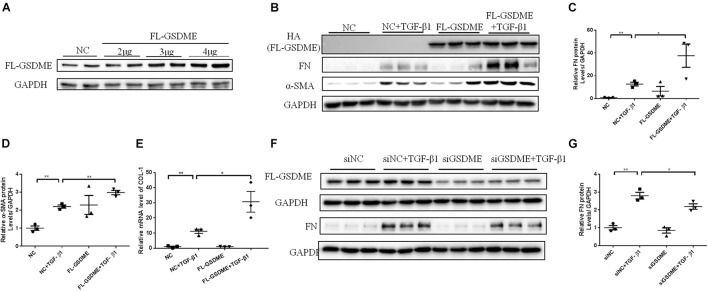
Full Length Gasdermin E (FL-GSDME) promotes profibrotic response in TGF-β1-treated HK-2 cells. HK-2 cells were treated with FL-GSDME overexpressing plasmid. **(A)** Transfection efficiency of FL-GSDME overexpressing plasmid at a dosage of 2, 3, and 4 μg/mL detected by western blotting analysis. **(B–D)** Western blotting analysis of FN and α-SMA in cells transfected with FL-GSDME plasmids (3 μg/mL) followed by treatment with TGF-β1 (10 ng/mL). **(E)** qPCR analysis of COL-1 in cells transfected with FL-GSDME plasmids (3 μg/ml) followed by treatment with TGF-β1 (10 ng/mL). HK-2 cells were treated with GSDME siRNA. **(F,G)** Silencing efficiency of FL-GSDME siRNA transfection detected by western blotting analysis. Western blotting analysis of FN in cells transfected with GSDME siRNAs followed by treatment with TGF-β1 (10 ng/mL). All the cell experiments, three independent experiments were performed. The quantitative results were shown as the means ± SEM. *n* = 3 in each group. *p < 0.05; **p < 0.01.

To further validate the role of GSDME in renal fibrosis, we first overexpressed FL-GSDME in HK-2 cells. After assessing the transfection efficiency of FL-GSDME by measuring the expression of HA-tag protein (Figure 3A), the regulatory effect of FL-GSDME on profibrotic response was evaluated. FL-GSDME overexpression significantly aggravated α-SMA, FN and COL-I expressions at both protein and mRNA levels (Figures 3B–E). Furthermore, we silenced *GSDME* in HK-2 cells by transfecting with *GSDME* siRNA (Figure 3F). TGF-β1 induced elevated FN protein was suppressed in *GSDME* knock-down cells (Figures 3F,G). These results provide strong evidence that GSDME plays a critical role in regulating the synthesis of ECM proteins in renal tubular cells and facilitate renal fibrosis deterioration.

### Overexpression of N-Gasdermin E Exacerbates Renal Fibrosis in Unilateral Ureteral Ligation Mice and TGF-β1-Treated Renal Tubular Epithelial Cells

**FIGURE 4:**
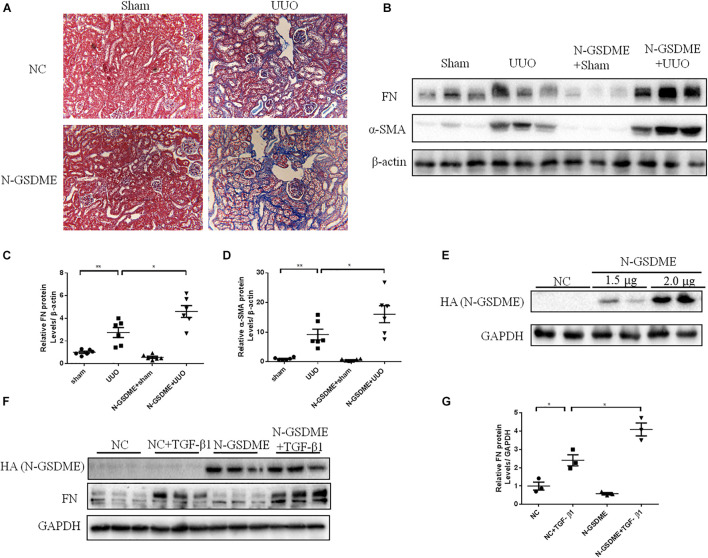
N-GSDME-induced pyroptosis exacerbates renal fibrosis in UUO mice and TGF-β1-treated renal tubular epithelial cells. Mice treated with N-GSDME overexpressing plasmid by tail vein injection and HK-2 cells treated with N-GSDME were used to determine the effect of N-GSDME in renal fibrosis. **(A)** Masson staining of kidney sections of N-GSDME overexpressing UUO mice (200×). **(B–D)** Western blotting analysis of FN and α-SMA in mouse kidneys from different groups (*n* = 6). **(E)** Transfection efficiency of N-GSDME overexpressing plasmid at a dosage of 0, 1.5, and 2 μg/mL detected by Western blotting analysis. **(F,G)** Western blotting analysis of FN in cells transfected with N-GSDME plasmids (2 μg/mL) followed by treatment with TGF-β1 (10 ng/mL), *n* = 3 in each group. For the cell experiments, three independent experiments were performed. The quantitative results were shown as the means ± SEM.*p < 0.05; **p < 0.01.

Full length gasdermin E is reportedly cleaved into N-GSDME to initiate pyroptosis and enhance the downstream reactions. To better explore whether GSDME plays a role in tubulointerstitial fibrosis through its N-terminal domain, we overexpressed N-*GSDME* plasmids into mice by tail vein injection followed by UUO. As expected, UUO-induced ECM deposition in the kidneys was significantly aggravated in mice overexpressing N-GSDME (Figure 4A). Similar results were obtained by detecting FN and α-SMA levels in N-GSDME overexpressing UUO kidneys compared with those in mice injected with empty vectors (Figures 4B–D). Meanwhile, we overexpressed N-GSDME plasmids in HK-2 cells (Figure 4E). Consistent with the results *in vivo*, upregulated N-GSDME also enhanced FN expression at the protein level (Figures 4F,G). These results indicate that N-GSDME-induced pyroptosis may be a pathogenic mechanism that exacerbates renal fibrosis.

### Inhibiting or Silencing Caspase-3 Alleviates Renal Fibrosis in Unilateral Ureteral Ligation Mice and TGF-β1-Treated Renal Tubular Epithelial Cells

**FIGURE 5:**
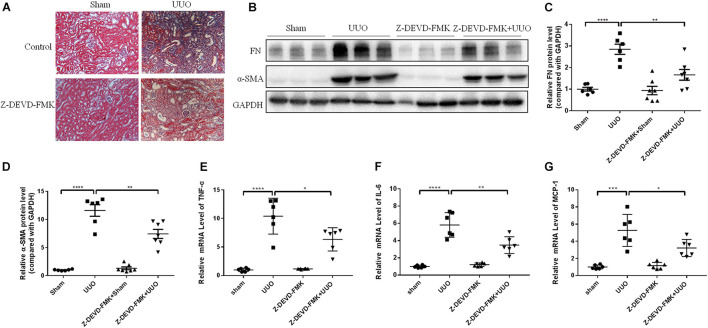
Caspase-3 inhibitor alleviates renal fibrosis in UUO mice. Mice were intraperitoneally injected with Z-DEVD-FMK to identify the effect of caspase-3 inhibition in renal fibrosis. **(A)** Masson staining of kidney sections of UUO mice with Z-DEVD-FMK intraperitoneal injection (200×). **(B–D)** Western blotting analysis of FN and α-SMA in mouse kidneys from different groups (*n* = 6–7). **(E–G)** qPCR analysis of tumor necrosis factor-alpha (TNF-α), IL-6, and MCP-1 in mouse kidneys from different groups (*n* = 6). All the quantitative results were shown as the means ± SEM. *p < 0.05; **p < 0.01; ***p < 0.001; ****p < 0.0001.

**FIGURE 6:**
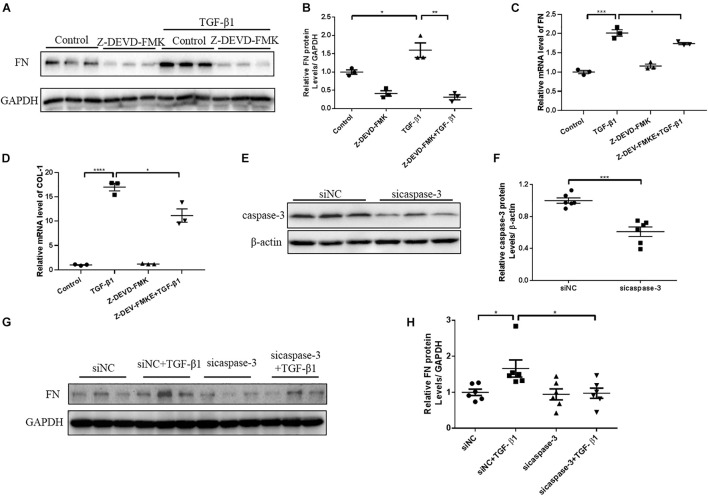
Caspase-3 inhibition alleviates profibrotic response in TGF-β1-treated renal tubular epithelial cells. HK-2 cells pretreated with Z-DEVD-FMK (100 μM) and cells treated with caspase-3 siRNA were used to identify the effect of caspase-3 inhibition on renal fibrosis. **(A,B)** Western blotting analysis of FN in HK-2 cells pretreated with Z-DEVD-FMK. **(C,D)** qPCR analysis of FN and COL-1 in HK-2 cells pretreated with Z-DEVD-FMK. **(E,F)** Silencing efficiency of caspase-3 siRNA detected by western blotting analysis. **(G,H)** Western blotting analysis of FN in cells transfected with caspase-3 siRNA followed by treatment with TGF-β1 (10 ng/mL). For the cell experiments, three independent experiments were performed. The quantitative results were shown as the means ± SEM. *n* = 3–6 in each group. *p < 0.05; **p < 0.01; ***p < 0.001; ****p < 0.0001.

N-GSDME-mediated pyroptosis is reportedly triggered by activated caspase-3 (Rogers et al., 2017). Considering the increased expression of cleaved caspase-3 in kidneys subjected to UUO, we further explored whether inhibiting or silencing caspase-3 could also modulate UUO-induced renal fibrosis. We first treated mice with Z-DEVD-FMK, a pharmacological inhibitor of caspase-3, following UUO. The pharmacological inhibition of caspase-3 blunted UUO-induced tubulointerstitial fibrosis and ECM protein synthesis (Figures 5A–D). Decreased inflammatory responses were observed in the kidneys of Z-DEVD-FMK treated mice, as evidenced by lower TNF-α, IL-6, and MCP-1 mRNA levels (Figures 5E–G). The anti-fibrotic effect of caspase-3 inhibition was further validated in HK-2 cells by both genetic silencing and pharmacological inhibition of caspase-3, as evidenced reduced FN and COL-1 mRNA expression (Figures 6A–H).

### *Gasdermin E* Deletion Ameliorates Renal Fibrosis and Inflammatory Responses in Kidneys Subjected to 5/6 Nephrectomy

**FIGURE 7:**
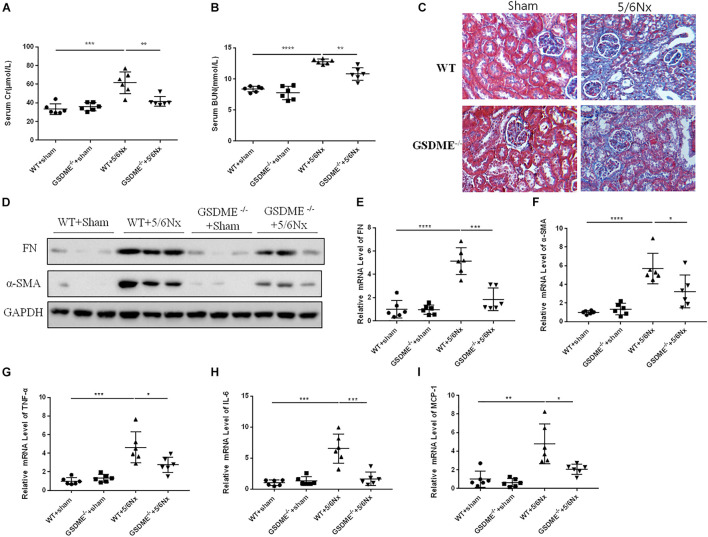
Gasdermin E deletion ameliorates renal fibrosis and inflammatory responses in kidneys challenged with 5/6 nephrectomy (5/6Nx). GSDME KO mice and WT mice were used to determine the role of GSDME in kidneys challenged with 5/6Nx. **(A,B)** Serum creatinine (CR) and blood urea nitrogen (BUN) levels of *GSDME* KO mice and controls (*n* = 6). **(C)** Masson staining of kidney sections of GSDME KO mice and WT mice (400×). **(D)** Western blotting analysis of FN and α-SMA in mouse kidneys from different groups (*n* = 3). **(E,F)** qPCR analysis of FN and α-SMA in mouse kidneys from different groups (*n* = 6). **(G–I)** qPCR analysis of TNF-α, IL-6, and MCP-1 in mouse kidneys from different groups (*n* = 6). The quantitative results were shown as the means ± SEM. *n* = 6 in each group. *p < 0.05; **p < 0.01; ***p < 0.001;****p < 0.0001.

Regarding the pathogenic role of GSDME in UUO-induced renal fibrosis and inflammation, we further evaluated the role of GSDME in another CKD model. Notably, 5/6Nx model is a well-established model of progressive CKD and is manifested by obvious renal tubulointerstitial fibrosis and renal dysfunction. We used *GSDME*-KO to determine the role of GSDME in 5/6Nx-induced kidney injury. *GSDME* deletion significantly reduced serum levels of CR and BUN and inhibited pathologic renal injury in mice with 5/6Nx (Figures 7A,B). Meanwhile, the renoprotective effect of *GSDME* deletion was also correlated with decreased renal interstitial fibrosis, which manifested as reduced α-SMA and FN expression (Figures 7C–F). Consistently, reduced inflammatory cytokine production was also observed in *GSDME* deficient mice suffered from 5/6Nx (Figures 7G–I). These data provide convincing evidence that GSDME may be a common target for treating different types of CKD.

## Discussion

Renal tubulo-interstitial fibrosis is considered the primary cause of CKD to ESRD, regardless of the initial causative factors (Chevalier, 2016). The degree of renal interstitial fibrosis is closely associated with renal function loss, and determines the curative effect (Meng et al., 2014). Consequently, promising anti-fibrotic therapeutic therapies are urgently needed for CKD.

Recent studies have determined that pyroptosis is a contributor for renal inflammation and injury in some acute kidney diseases (Miao et al., 2019). In this study, we explored whether the regulation of pyroptosis might be helpful in delaying the progression of renal fibrosis and functional loss in CKD. Previous studies showed that activation of NLRP3 inflammasomes in UUO and 5/6Nx kidneys was accompanied by pyroptosis at the early stage of tubulo-interstitial fibrosis. *NLRP3* KO contributes to decreased caspase-1, IL-1β, and IL-18 production in the kidneys of UUO mice, accompanied by reduced tubulointerstitial fibrosis (Guo et al., 2017). However, owing to the multiple inflammatory cascade reaction of inflammasome activation, these studies based on NLRP3 and caspase-1 could not provide direct evidence for the involvement of pyroptosis in CKD progression.

Gasdermin E, another key protein in addition to GSDMD, has been recently found to mediate pyroptosis through its N-terminal domain, which can oligomerize and form a membrane pore to release cellular inflammatory cytokines (Wang et al., 2017). Another recent study has shown that GSDME-mediated cell death may be involved in the development of diabetic nephropathy (Wen et al., 2020). Moreover, a parallel study showed the anti-fibrotic effect of *GSDME* deletion in obstructive nephropathy (Li et al., 2021). However, owing to the limitations of the UUO model, whether GSDME contributes to the pathogenesis of other types of CKD and renal dysfunction remains to be investigated. In addition, it is of importance to evaluate the role of active N-GSDME in CKD animals because the FL-GSDME could play roles in the experimental setting *via* an N-GSDME-independent mechanism. Therefore, studies targeting GSDME in different CKD models and manipulating N-GSDME in animals will provide more valuable evidence to better clarify the role of GSDME and N-GSDME in CKD.

In this study, we detected an increased expression of N-GSDME in UUO kidneys, accompanied by increased cleaved caspase-3 expression, and IL-1β production. These data indicate that GSDME-mediated pyroptosis may participate in UUO-induced kidney damage. Next, we applied GSDME KO mice to verify the role of GSDME in renal interstitial fibrosis. *GSDME* KO alleviated UUO-induced ECM synthesis. Accumulating evidence has indicated a close link between inflammation and CKD. The unresolved inflammatory response is the main driving force in the development of fibrotic diseases (Nathan and Ding, 2010). Induced synthesis and release of IL-1β was thought to lead to further inflammatory cell recruitment and activation (Inoue et al., 2015). In the present study, we also found that GSDME deletion exerted a renal protective effect in 5/6Nx CKD along with attenuated renal fibrosis, inflammation and renal dysfunction, which highlights that GSDME might contribute to the pathogenesis of different types of CKD via promoting inflammation.

TGF-β is a key factor in the development of renal fibrosis (Fan et al., 1999). Upon treatment with TGF-β1, renal tubular epithelial cells always exhibit cellular phenotypic changes and induce ECM synthesis. In TGF-β1-treated HK-2 cells, we detected an enhanced expression of N-GSDME, which suggested that GSDME-mediated pyroptosis in renal tubular epithelial cells might be responsible for the progression of UUO-induced kidney injury. In addition, using the genetic overexpression approach, the pro-fibrogenic effect of GSDME N-terminal domain was observed in UUO kidneys and HK-2 cells, which is consistent with FL-GSDME overexpression in cells. These findings suggest that the pro-fibrosis effect of GSDME is mediated by N-GSMDE and pyroptosis.

Pyroptosis is a type of programmed cell death and is dependent on activation of different caspases. Caspase-3 cleaves GSDME in cells to release the N-terminal domain and induce pyroptosis, which shifts the concept that caspase-3 activation inevitably leads to apoptosis. Recent studies have shown that caspase-3 inhibitor, Z-DEVD-FMK, attenuates renal interstitial fibrosis in diabetic nephropathy (Wen et al., 2020). In this present study, we used caspase-3 inhibitor, Z-DEVD-FMK, in UUO model and TGF-β1 treated HK-2 cells and observed an antifibrotic effect of caspase-3 inhibition, which was consistent with the findings in recent study by Li et al. (2021).

Recent research reported that granzyme B could activate caspase-independent pyroptosis by directly cleaving GSDME at D270 (Zhang et al., 2020). Previous findings and our data (not shown) showed that the expression of granzyme B in fibrotic kidney was significantly upregulated (Law et al., 2019). Thus, we cannot rule out the contribution of granzyme B in inducing GSDME-dependent pyroptosis in CKDs by cleaving GSDME. Therefore, further studies are required to define the role of granzyme B on activating GSDME in CKDs.

In summary, GSDME can participate in renal tubulointerstitial fibrosis and renal dysfunction in CKDs through N-GSDME-dependent pyroptosis. Our findings provide insights into GSDME-mediated pyroptosis as a new potential target for the prevention and treatment of CKDs.

## Data Availability Statement

The original contributions presented in the study are included in the article/supplementary material, further inquiries can be directed to the corresponding authors.

## Ethics Statement

The animal study was reviewed and approved by Nanjing Medical University Institutional Animal Care and Use Committee.

## Author Contributions

ZJ, YZ, and YL conceived of and supervised the research. MW, YL, WX, QJ, AnZ, and QW designed, performed, analyzed most of the experiments, and drafted the manuscript. SH and AiZ provided the technical advices. All authors contributed to manuscript revision and approved the submitted version.

## Conflict of Interest

The authors declare that the research was conducted in the absence of any commercial or financial relationships that could be construed as a potential conflict of interest.

## Publisher’s Note

All claims expressed in this article are solely those of the authors and do not necessarily represent those of their affiliated organizations, or those of the publisher, the editors and the reviewers. Any product that may be evaluated in this article, or claim that may be made by its manufacturer, is not guaranteed or endorsed by the publisher.

## References

[B1] Chen X. He W. T. Hu L. Li J. Fang Y. Wang X. (2016). Pyroptosis is driven by non-selective gasdermin-D pore and its morphology is different from MLKL channel-mediated necroptosis. *Cell Res.* 26 1007–1020. 10.1038/cr.2016.100 27573174 PMC5034106

[B2] Chevalier R. L. (2016). The proximal tubule is the primary target of injury and progression of kidney disease: role of the glomerulotubular junction. *Am. J. Physiol. Renal. Physiol.* 311 F145–F161. 10.1152/ajprenal.00164.2016 27194714 PMC4967168

[B3] Ding J. Wang K. Liu W. She Y. Sun Q. Shi J. (2016). Pore-forming activity and structural autoinhibition of the gasdermin family. *Nature* 535 111–116. 10.1038/nature18590 27281216

[B4] Fan J. M. Ng Y. Y. Hill P. A. Nikolic-Paterson D. J. Mu W. Atkins R. C. (1999). Transforming growth factor-beta regulates tubular epithelial-myofibroblast transdifferentiation in vitro. *Kidney Int.* 56 1455–1467. 10.1046/j.1523-1755.1999.00656.x 10504497

[B5] Gluck C. Qiu C. Han S. Y. Palmer M. Park J. Ko Y. A. (2019). Kidney cytosine methylation changes improve renal function decline estimation in patients with diabetic kidney disease. *Nat. Commun.* 10:2461. 10.1038/s41467-019-10378-8 31165727 PMC6549146

[B6] Gong W. Mao S. Yu J. Song J. Jia Z. Huang S. (2016). NLRP3 deletion protects against renal fibrosis and attenuates mitochondrial abnormality in mouse with 5/6 nephrectomy. *Am. J. Physiol. Renal. Physiol.* 310 F1081–F1088. 10.1152/ajprenal.00534.2015 26887832

[B7] Guo H. Bi X. Zhou P. Zhu S. Ding W. (2017). NLRP3 deficiency attenuates renal fibrosis and ameliorates mitochondrial dysfunction in a mouse unilateral ureteral obstruction model of chronic kidney disease. *Mediators Inflamm.* 2017:8316560. 10.1155/2017/8316560 28348462 PMC5350413

[B8] Hill N. R. Fatoba S. T. Oke J. L. Hirst J. A. O’Callaghan C. A. Lasserson D. S. (2016). Global prevalence of chronic kidney disease – a systematic review and meta-analysis. *PLoS One* 11:e0158765. 10.1371/journal.pone.0158765 27383068 PMC4934905

[B9] Inoue T. Umezawa A. Takenaka T. Suzuki H. Okada H. (2015). The contribution of epithelial-mesenchymal transition to renal fibrosis differs among kidney disease models. *Kidney Int.* 87 233–238. 10.1038/ki.2014.235 25007169

[B10] Law B. M. P. Wilkinson R. Wang X. Kildey K. Giuliani K. Beagley K. W. (2019). Human tissue-resident mucosal-associated invariant T (MAIT) cells in Renal fibrosis and CKD. *J. Am. Soc. Nephrol.* 30 1322–1335. 10.1681/ASN.2018101064 31186283 PMC6622420

[B11] Levey A. S. Atkins R. Coresh J. Cohen E. P. Collins A. J. Eckardt K. U. (2007). Chronic kidney disease as a global public health problem: approaches and initiatives – a position statement from Kidney Disease Improving Global Outcomes. *Kidney Int.* 72 247–259. 10.1038/sj.ki.5002343 17568785

[B12] Li X. Zeng L. Cao C. Lu C. Lian W. Han J. (2017). Long noncoding RNA MALAT1 regulates renal tubular epithelial pyroptosis by modulated miR-23c targeting of ELAVL1 in diabetic nephropathy. *Exp. Cell Res.* 350 327–335. 10.1016/j.yexcr.2016.12.006 27964927

[B13] Li Y. Xia W. Wu M. Yin J. Wang Q. Li S. (2020). Activation of GSDMD contributes to acute kidney injury induced by cisplatin. *Am. J. Physiol. Renal. Physiol.* 318 F96–F106. 10.1152/ajprenal.00351.2019 31682173

[B14] Li Y. Yuan Y. Huang Z. X. Chen H. Lan R. Wang Z. (2021). GSDME-mediated pyroptosis promotes inflammation and fibrosis in obstructive nephropathy. *Cell Death Differ.* 28 2333–2350. 10.1038/s41418-021-00755-6 33664482 PMC8329275

[B15] Lu H. Zhang S. Wu J. Chen M. Cai M. C. Fu Y. (2018). Molecular targeted therapies elicit concurrent apoptotic and gsdme-dependent pyroptotic tumor cell death. *Clin. Cancer Res.* 24 6066–6077. 10.1158/1078-0432.CCR-18-1478 30061362

[B16] Mamik M. K. Power C. (2017). Inflammasomes in neurological diseases: emerging pathogenic and therapeutic concepts. *Brain* 140 2273–2285. 10.1093/brain/awx133 29050380

[B17] Meng X. M. Nikolic-Paterson D. J. Lan H. Y. (2014). Inflammatory processes in renal fibrosis. *Nat. Rev. Nephrol.* 10 493–503. 10.1038/nrneph.2014.114 24981817

[B18] Miao N. Yin F. Xie H. Wang Y. Xu Y. Shen Y. (2019). The cleavage of gasdermin D by caspase-11 promotes tubular epithelial cell pyroptosis and urinary IL-18 excretion in acute kidney injury. *Kidney Int.* 96 1105–1120. 10.1016/j.kint.2019.04.035 31405732

[B19] Nathan C. Ding A. (2010). Nonresolving inflammation. *Cell* 140 871–882. 10.1016/j.cell.2010.02.029 20303877

[B20] Orning P. Lien E. Fitzgerald K. A. (2019). Gasdermins and their role in immunity and inflammation. *J. Exp. Med.* 216 2453–2465. 10.1084/jem.20190545 31548300 PMC6829603

[B21] Peng X. Yang T. Liu G. Liu H. Peng Y. He L. (2018). Piperine ameliorated lupus nephritis by targeting AMPK-mediated activation of NLRP3 inflammasome. *Int. Immunopharmacol.* 65 448–457. 10.1016/j.intimp.2018.10.025 30388519

[B22] Rogers C. Fernandes-Alnemri T. Mayes L. Alnemri D. Cingolani G. Alnemri E. S. (2017). Cleavage of DFNA5 by caspase-3 during apoptosis mediates progression to secondary necrotic/pyroptotic cell death. *Nat. Commun.* 8:14128. 10.1038/ncomms14128 28045099 PMC5216131

[B23] Schnaper H. W. (2017). The tubulointerstitial pathophysiology of progressive kidney disease. *Adv. Chronic Kidney Dis.* 24 107–116. 10.1053/j.ackd.2016.11.011 28284376 PMC5351778

[B24] Shi J. Zhao Y. Wang K. Shi X. Wang Y. Huang H. (2015). Cleavage of GSDMD by inflammatory caspases determines pyroptotic cell death. *Nature* 526 660–665. 10.1038/nature15514 26375003

[B25] Wang Y. Gao W. Shi X. Ding J. Liu W. He H. (2017). Chemotherapy drugs induce pyroptosis through caspase-3 cleavage of a gasdermin. *Nature* 547 99–103. 10.1038/nature22393 28459430

[B26] Wen S. Wang Z. H. Zhang C. X. Yang Y. Fan Q. L. (2020). Caspase-3 promotes diabetic kidney disease through gasdermin E-mediated progression to secondary necrosis during apoptosis. *Diabetes Metab. Syndr. Obes.* 13 313–323. 10.2147/DMSO.S242136 32104028 PMC7020918

[B27] Wu H. Huang T. Ying L. Han C. Li D. Xu Y. (2016). MiR-155 is involved in renal ischemia-reperfusion injury via direct targeting of FoxO3a and regulating renal tubular cell pyroptosis. *Cell. Physiol. Biochem.* 40 1692–1705. 10.1159/000453218 28006785

[B28] Xia W. Li Y. Wu M. Jin Q. Wang Q. Li S. (2021). Gasdermin E deficiency attenuates acute kidney injury by inhibiting pyroptosis and inflammation. *Cell Death Dis.* 12:139. 10.1038/s41419-021-03431-2 33542198 PMC7862699

[B29] Yang J. R. Yao F. H. Zhang J. G. Ji Z. Y. Li K. L. Zhan J. (2014). Ischemia-reperfusion induces renal tubule pyroptosis via the CHOP-caspase-11 pathway. *Am. J. Physiol. Renal. Physiol.* 306 F75–F84. 10.1152/ajprenal.00117.2013 24133119

[B30] You R. Zhou W. Li Y. Zhang Y. Huang S. Jia Z. (2020). Inhibition of ROCK2 alleviates renal fibrosis and the metabolic disorders in the proximal tubular epithelial cells. *Clin. Sci.* 134 1357–1376. 10.1042/CS20200030 32490513

[B31] Zhang Z. Shao X. Jiang N. Mou S. Gu L. Li S. (2018). Caspase-11-mediated tubular epithelial pyroptosis underlies contrast-induced acute kidney injury. *Cell. Death Dis.* 9:983. 10.1038/s41419-018-1023-x 30250284 PMC6155357

[B32] Zhang Z. Zhang Y. Xia S. Kong Q. Li S. Liu X. (2020). Gasdermin E suppresses tumour growth by activating anti-tumour immunity. *Nature* 579 415–420. 10.1038/s41586-020-2071-9 32188940 PMC7123794

[B33] Zheng X. Zhong T. Ma Y. Wan X. Qin A. Yao B. (2020). Bnip3 mediates doxorubicin-induced cardiomyocyte pyroptosis via caspase-3/GSDME. *Life Sci.* 242:117186. 10.1016/j.lfs.2019.117186 31862454

